# Association of food insecurity with dietary patterns and expenditure on food, alcohol and tobacco amongst indigenous Inuit in Greenland: results from a population health survey

**DOI:** 10.1186/s12889-021-11123-x

**Published:** 2021-06-08

**Authors:** Peter Bjerregaard, Ingelise Olesen, Christina Viskum Lytken Larsen

**Affiliations:** 1 Centre for Public Health in Greenland, National Institute of Public Health, University of Southern Denmark, Studiestræde 6, 1455 Copenhagen K, Denmark; 2 Institute for Nursing and Health Research, University of Greenland, P.0. Box 1061, Manutooq 1, 3905 Nuussuaq, Greenland

**Keywords:** Food insecurity, Dietary patterns, Commodity basket cost, Alcohol, Tobacco, Inuit, Greenland

## Abstract

**Background:**

Amongst the indigenous Greenlandic Inuit, the experience of food insecurity has been attributed to a lack of money to buy enough food of sufficient quality to sustain a family, although a preference for alcohol and tobacco over food has also been cited. The purpose of the article was to compare dietary patterns and expenditure on food, alcoholic beverages and tobacco between survey participants who reported food insecurity and those who did not.

**Methods:**

A countrywide cross-sectional health survey was carried out among 1886 adult Greenlandic Inuit in 2018. Diet was estimated by a food frequency questionnaire. Food insecurity status was based on the household hunger scale. Analyses were carried out by univariate general linear models adjusted for age, sex and social position.

**Results:**

Nine percent of the participants reported food insecurity. Food insecurity was higher among younger participants, men and participants with low social position. Food insecure participants more often chose an unhealthy dietary pattern (43% vs. 32%) and they reported a higher energy intake. The food insecure spent the same amount of money on food as other participants but less on nutritious food and more on non-nutritious food. The cost per kilojoule (kJ) of the food of the food insecure was lower than that of the food secure (DKK 8.0 and 9.0 per 1000 kJ, respectively). The food insecure participants also spent considerably more on alcohol and tobacco.

**Conclusions:**

The results suggest that it is not only unemployment and lack of money that creates food insecurity and unhealthy dietary patterns in Greenland. Food insecure participants gave higher priority to buying non-nutritious food, alcohol and tobacco than did food secure participants. There seems to be at least two population subgroups in Greenland with poverty and substance use, respectively, as the immediate determinants for food insecurity. The results are important for the design of interventions against food insecurity and unhealthy dietary patterns.

**Supplementary Information:**

The online version contains supplementary material available at 10.1186/s12889-021-11123-x.

## Background

Food insecurity has many aspects worldwide and in the high North [1]. According to a generally accepted definition, food security is ‘adequate access to food for all people at all times for an active, healthy life’ [2]. Food also has an important social role in keeping families and communities together. Food insecurity is a worldwide problem among poor, marginalized and indigenous populations ranging from inability to access culturally desirable food to hunger and starvation [2]. Even among the Greenlandic Inuit, who live in a modern welfare state, food insecurity is present, and is partly due to constrained access to *kalaalimernit* (locally harvested food) [3]. The theoretical framework for our studies of food insecurity builds on the concepts of availability and accessibility of food [4]. Availability is provided by the food system – market and local sources – that place food at the disposal of consumers. Accessibility is the financial and social resources of individuals to acquire the available food. Food insecurity occurs when the balance between needs and resources is upset. In the present paper, our focus is on socioeconomic resources and expenditure on alcohol and tobacco as possible determinants of food insecurity and on dietary patterns as a possible result of food security status. However, in a cross-sectional study the direction of causality cannot be ascertained.

Greenland is an autonomous part of the Kingdom of Denmark. Greenland has approximately 80 communities (towns and villages) situated mostly on the Central West Coast with no connecting roads. The climate is arctic. A town is defined historically as the largest community in each of 17 districts. In 2018, the population of the towns varied between 366 and 5491, and an additional 17,796 residents lived in Nuuk, the capital. The population in villages varied from less than 10 to approximately 550. The total population of Greenland is 56,000, of whom 92% are Greenlandic Inuit (syn. Greenlanders, Kalaallit) [5]. Genetically, Greenlanders are Inuit with a 25% admixture of European, mainly Scandinavian, genes [6]. Greenlandic Inuit are closely related genetically and culturally to the Inuit/Iñupiat/Inuvialuit in Canada and Alaska and somewhat more distantly to the Yupiit of Alaska and Siberia [7]. In Greenland, approximately 80% of food is imported food items purchased in kiosks or supermarkets, whereas locally harvested food is either purchased at local hunters’ markets or hunted and fished privately [8, 9]. In the villages, the shops have less food available than in towns. Alcohol and tobacco are also sold in supermarkets.

The prevalence of food insecurity varies among European and North American countries, but so do the methods for assessment, and comparisons at international level is problematic. Most studies, however, agree that low income and poor social conditions are strong predictors of food insecurity [10–14]. After socio-demographic adjustment, adults from food insecure households in Denmark had a higher probability of eating an unhealthy diet [12]. In France, no significant difference between food insecure and other survey participants was found for energy intake, but the intake of fruits, vegetables and fish was lower, and diet quality was poorer among the food insecure, who also spent less money on food [10].

Among indigenous peoples in affluent Western countries, food insecurity is considered a public health issue, and in particular a high prevalence has been described among the Inuit in Canada [15–17]. Low formal education attainment and unemployment were associated with increased odds of food insecurity in households with children. Fruit and vegetable consumption, as well as eating cooked or raw fish, was associated with decreased odds of food insecurity, whereas eating frozen meat and fish was associated with increased odds of food insecurity [18]. Poor socioeconomic conditions, in particular household crowding, increased the likelihood of food insecurity among children [19]. In contrast to Canada, there is limited research available on food security among Alaska Natives [20].

Few studies of food security have been carried out in Greenland. Rasmussen and co-workers synthesised the issue at a macro level with emphasis on access to Greenlandic food (*kalaalimernit*) [9]. A study from a small town in West Greenland concluded that 8% of the population were food insecure and that access to the culturally significant *kalaalimernit* was limited among women, adults aged 55+ and non-hunters [3]. Among Greenlandic school children aged 11–17 years, 13% reported always or often going to bed or to school hungry because of lack of food at home [21].

Further studies from Greenland showed that the prevalence of food insecurity decreased from 2014 to 2018 (relative risk 0.69; 95% confidence interval 0.57–0.85) [22]. Food insecurity also varied significantly among regions. In East and North Greenland, 21% reported food insecurity, compared with 7% in West Greenland (*p* < 0.0001). In West Greenland, food insecurity was higher in villages on the southwest coast than in other towns and villages. Food security varied significantly by wealth. Among the poorest one-fifth of the participants, food insecurity was reported at 20%. Among the wealthiest one-fifth, only 0.5% reported food insecurity [23]. Some of the geographic variation disappeared after adjustment for individual wealth or employment [22].

In 2016 and 2017, nineteen qualitative interviews were conducted in Nuuk, the capital of Greenland, and Tasiilaq, a small town on the remote East Coast, among participants in a population health survey, who in 2014 had reported food insecurity [22]. Especially unemployed participants and families subsisting on minimum wages perceived the main cause of food insecurity to be a lack of money and high cost of nutritious food and *kalaalimernit*. The main meal of the day was reported to consist of cheap food like breakfast cereal, pancakes or plain pasta for the children, whereas the parents ate very little or went hungry. Several participants admitted that the purchase of alcohol and tobacco instead of food contributed to food insecurity. A recent study from Greenland showed that participants with low social position spent less money on the total food basket than those with high social position but more money on non-nutritious food and considerably more money on alcohol and tobacco [24].

There are numerous studies on the association between social position and health behaviour [25]. A review of socioeconomic disparities in health behaviour outlined nine possible explanations of more smoking, less exercise, poorer diet and excess weight among persons with low socioeconomic status [26]. The explanations were related but conceptually distinct. For Greenland, in particular deprivation, efficacy and agency, and knowledge are relevant themes.

The aim of the article is to explore the association of food insecurity with dietary patterns and the expenditure on food, alcoholic beverages and tobacco. Specifically, it is examined if the observations from a qualitative study of food insecurity in Greenland can be confirmed, namely that food insecurity is primarily due to lack of money and high cost of nutritious food, and that in some cases purchase of alcohol and tobacco is prioritised over purchase of food [22]. It is hypothesised that after adjustment for social position, those who report food insecurity have less energy intake than other survey participants and eat less healthy food, in particular less *kalaalimernit* other than fish, fruit and vegetables, which are all rather expensive, and spend less money on food. Based on previous research from Greenland, it is further hypothesised that expenditure on alcohol and tobacco is higher among the food insecure than among food secure Inuit of equal social position, thus contributing to the experience of food insecurity.

## Methods

### Data collection

**Fig. 1:**
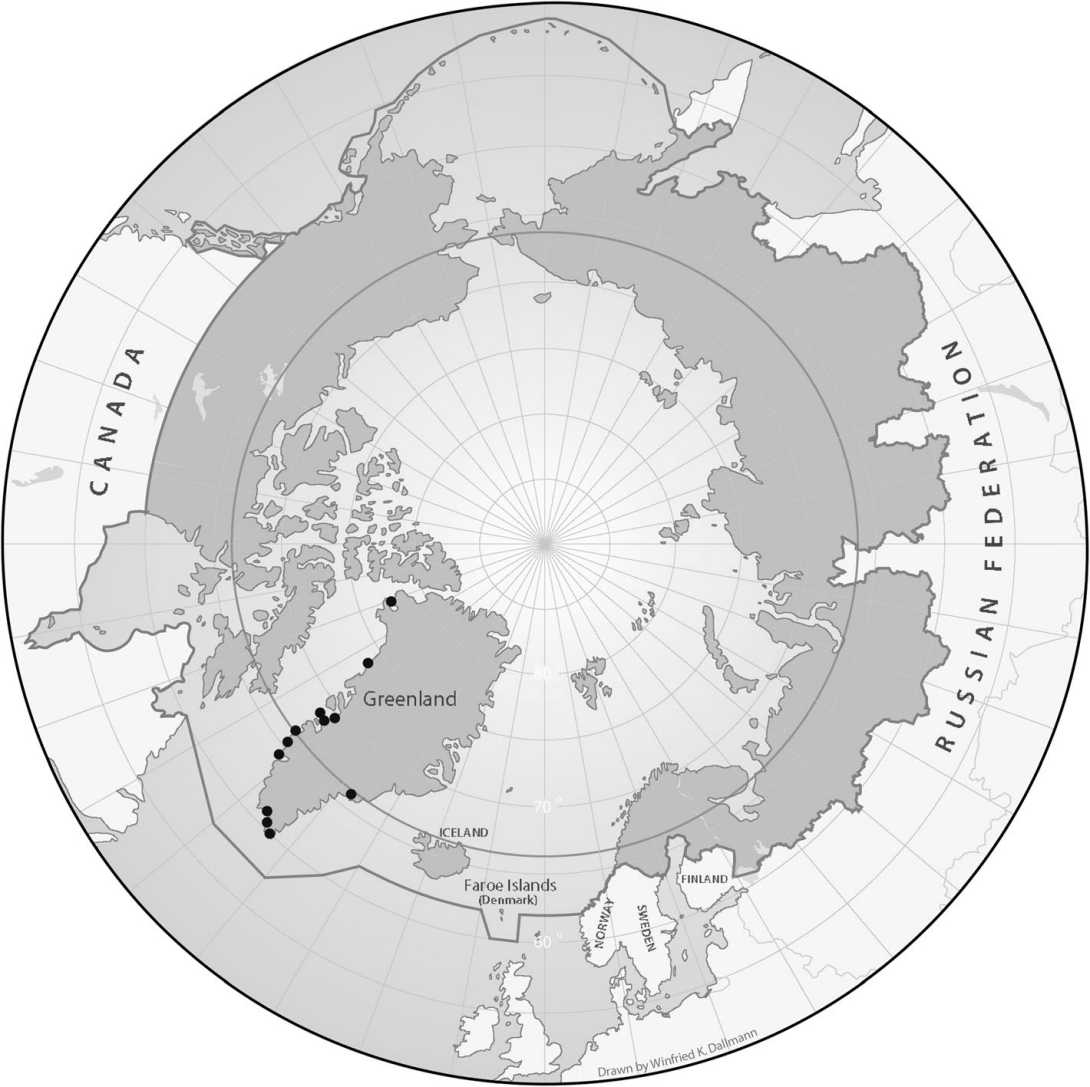
Map of the circumpolar region with data collection sites in Greenland marked with black circles. Map by Winfried Dallmann, Norwegian Polar Centre

Data were collected from 2017 to 2019 as part of a countrywide cross-sectional health survey in Greenland (Fig. 1) [23]. The participants, aged 15 years and older, were selected through a stratified random sample of adults in Greenland, who had been born in Greenland or Denmark. From each of five strata defined according to geographical criteria (South, Mid, Northwest, North and East Greenland), 20 towns and villages were chosen at random. From 12 towns, a random sample of inhabitants aged 15+ years were invited and from 8 villages all inhabitants were invited to participate in the study. Data were collected by interviews and self-administered questionnaires. The participation rate was 52%. Interviews provided information about socio-demographic factors, diet and smoking as further discussed below. Alcohol use was reported in the self-administered AUDIT questionnaire [27]. Questionnaires were developed in the Danish language, translated into Greenlandic (*kalaallisut*), back-translated and revised. Interviews were conducted in the participant’s language of choice, which was most often Greenlandic, by native Greenlandic speaking interviewers who had been trained in the study procedures. The mean duration of an interview was 49 min. A total of 2436 Inuit participated in the survey. Inuit ethnicity was defined by the interviewers at enrolment based on primary language and self-identification. Participants whose information on diet was considered not to be valid (see below) and participants living in villages - from where information on food prices was not obtained - were excluded reducing the number of participants to 1886.

### Demographic and social indicators

The household asset score is an indicator of social position that is closely associated with more traditional measures of socioeconomic position, such as education and income, but it has certain practical advantages and is often used in a Greenlandic context [28, 29]. Participants were asked about their ownership of six household items: video/DVD player, computer, microwave oven, washing machine, dishwashing machine and internet. Here, 0/1 answers were added, yielding a score ranging from 0 to 6. Participants with scores 0–2 were subsequently combined due to few participants in each category. All participants answered these questions. For participants aged 18+ years (*N* = 1830), education was recorded from survey information on school and occupational education into five categories according to the highest achievement: primary school; upper secondary school; short vocational education (1–2 years); medium long education (2–3 years); long education, university (4+ years). Information on education was available for 1773 participants (97%). Participants aged 18–65 years (*n* = 1505) were asked about their job title. The answers were manually recoded into six categories: not gainfully employed; students; unskilled workers; hunters, fishermen and assisting wives; skilled workers; white-collar employees. Information was available for 1473 participants (98%). The three variables that described social position were closely associated. Household asset score was included in the statistical models because it is defined for the whole population (unlike job category) and because it fitted a normal distribution better than both education and job category.

### Food intake and cost of food

The interview included a 47-item quantitative food frequency questionnaire with 13 locally harvested and 34 imported food items (Table 1). Interviewers were instructed to ask about typical food intake during the last 12 months. The frequency of consumption was reported as the (open-ended) number of times per day, week, month, or year. For each food item, one of four portion sizes was selected (fractions were allowed). The food frequency questionnaire has been validated by biomarkers for the traditional marine diet [30]. Food intake was estimated by multiplying frequency with portion sizes. The intake of energy and macronutrients was calculated from published concentrations [31, 32] using Microsoft Excel Office 365 and IBM SPSS Statistics version 26. The individual energy expenditure was estimated as the basal metabolic rate calculated from age, sex and weight [33] multiplied with the physical activity level (PAL) determined from questions about usual physical activity during summer and winter. PAL ranged from 1.3 to 1.9. Participants with a ratio between reported and calculated energy below the 2.5 percentile or above the 97.5 percentile or with a reported energy intake below 2100/3350 kJ or above 15,000/17,000 kJ for women/men [34] were excluded (*n* = 203; 8.3%). Dietary patterns were calculated according to a method described earlier [35] with a few revisions: The algorithm initially classified all participants as having a ‘standard diet’. If their diet contained 30 energy percent (E%) or greater from imported meat, participants were reclassified as having a dietary pattern of ‘imported meat’. In the next step, if 30 E% or greater came from food items categorised as unhealthy (sweets, sugar-sweetened beverages, cakes and cookies, added sugar, convenience snacks, fast food), participants were reclassified as having an ‘unhealthy diet’. In the next step, if their diet contained 30 E% or greater from locally harvested food, participants were reclassified as having a ‘*kalaalimernit* (local Greenlandic food)’ dietary pattern. Finally, participants who met eight or more of nine criteria for a balanced diet were reclassified as having a ‘balanced’ dietary pattern. The nine criteria were defined by operationalising the official dietary guidelines for Greenland [36]. The criteria for a balanced diet were as follows: greater than 10 E% *kalaalimernit* but consumption of marine mammals less often than 4 times a week; consumption of fish at least twice a week; consumption of fruit at least three times a week; consumption of vegetables at least three times a week; consumption of whole grain products at least three times a week; consumption of less than 35 E% fat; consumption of less than 15 E% refined sugar; consumption of crisps less often than daily; and drinking of sugar-sweetened beverages less often than daily. The algorithm implied that if a participant initially fell into more than one category, ‘balanced diet’ was chosen above the four other categories. Furthermore, ‘*kalaalimernit*’ was chosen above ‘unhealthy’, ‘imported meat’, and ‘standard diet’; ‘unhealthy’ was chosen above ‘imported meat’ and ‘standard diet’; and ‘imported meat’ was chosen above ‘standard diet’. Alcohol consumption was estimated as the product of the two first questions in the AUDIT questionnaire [28].

**Table 1:** Items in the food frequency questionnaire. Items marked with an * are considered non-nutritious

**Kalaalimernit** **(*N*** **= 13)**	
Seal meat	Fruit juice*
Whale meat	Mixed vegetables (fresh or frozen)
Muktuk (whale skin)	Potatoes
Cod	Carrots
Greenland halibut	Cabbage
Capelin	Tomatoes
Arctic char, salmon	Milk
Other fish	Cheese
Caribou and muskox meat	White bread
Game birds	Rye bread
Berries	Cereals
Dried fish or meat	Oat flakes, porridge
Blubber (frozen, salted)	Pasta
**Imported food (*N*** **= 34)**	Rice
Beef	Cakes, biscuits*
Pork	Sweets*
Mutton	Fizzy drinks*
Poultry	Fruit syrup*
Ready-made food (canned, frozen)	Pizza, burger*
Cold cuts	French fries*
Canned fish	Crisps*
Apples, pears, bananas	Sugar (in tea, coffee)*
Oranges, grapefruit	Tea*
Other fresh fruit	Coffee*

The prices of the 47 food items as well as alcoholic beverages and cigarettes were collected from 14 shops, including the two major food chains in Greenland (*Pisiffik/Pilersuisoq* and *Brugseni*) and some private kiosks in 7 of the 12 towns where interviews were conducted. Both normal prices and special offers were recorded. The interviewers who collected the prices were asked to choose from the many varieties of similar food at their own discretion. Although the collection of prices was not further standardised, it is believed that the variety of towns and shop types give a reasonably estimate of average prices. Average prices were calculated as arithmetic means. Analyses of the expenditure on food were only performed for residents of towns (82% of the sample) because the information about the cost of locally harvested food fluctuated considerably in the villages due to food sharing and informal markets and because information about the cost of store-bought food was only available from one village. Prices were calculated for each of the 47 individual food items and for nutritious and non-nutritious food groups. The latter comprised sweets, sugar-sweetened beverages, cakes and biscuits, added sugar, convenience snacks, fruit juice, tea and coffee. The commodity basket included food, alcoholic beverages and tobacco.

### Food security

Food security was assessed by three questions adapted from the household hunger scale [37], namely ‘In the past twelve months, was there ever no food to eat of any kind in your house because of a lack of resources to obtain food?’; ‘In the past twelve months, did you or any household member go to sleep at night hungry because there was not enough food?’; and ‘In the past twelve months, did you or any household member go a whole day and night without eating anything because there was not enough food?’ If a participant answered yes to one of the questions, food insecurity was present. The household hunger scale has three main questions about food insecurity during the past 4 weeks plus three questions about the number of times each of the three problems happened. In the course of adaptation for use in the survey in Greenland, the questions about number of times were left out and the time frame was changed from the past 4 weeks to the past 12 months. Questions were translated from English to Greenlandic and independently back-translated by skilled interpreters. In Arctic Canada, the use of a single item food security questionnaire has proven feasible [38] whereas in Greenland, the use of three instead of one question about food security slightly increased the proportion of food insecure participants (from 7.9 to 8.8%) [22]. Information was available for all participants.

### Statistical methods

Univariate general linear models were applied throughout with various dependent variables, food security as factor, and age and sex or age, sex and social position as covariates. The models provided estimates of means of the dependent variable for categories of the factor (food security) adjusted for the covariates. These estimates were tabulated in Tables 3, 4 and 5. We used the standard statistical programme IBM SPSS Statistics version 26. In Tables 3 and 5 we presented *p*-values as indicators of statistical significance of comparisons. In Table 4, ratios between adjusted proportions with 95% confidence intervals were calculated by the online statistics calculator GraphPad [39]. In Table 4, outcome variables (cost, energy) were tested by Q-Q plots. Normal distribution was assumed, and calculations were performed using untransformed values.

## Results

### Demography and social position

The data were geographically representative of all of Greenland and the participants comprised greater than 6% of the adult Inuit population. However, the cost of food, alcohol and tobacco was only available for the population in towns (82% of participants). Table 2 presents the population characteristics of all 2436 Inuit in the survey and the 1886 Inuit from towns with valid information on diet, who comprised the study base. With an age span of 15 to 94 years, the median age was 49 years (IQR (interquartile range) 35–61 years). Women were overrepresented compared with the population (55% vs. 49%) due to their higher participation rate. Household assets were normally distributed and slightly shifted to the right with an average score of 4.2. The 1886 participants in the study base were more affluent, had better education and held higher job position than all Inuit participants. This scenario reflects the socioeconomic difference between towns and villages. Nine percent of the sample reported food-insecurity.

**Table 2:** Population characteristics of all 2436 Greenland Inuit in the 2018 Greenland Population Health Survey and of 1886 urban Inuit with valid information on diet

	N	Median (IQR)	N	Median (IQR)
**Age (years)**	2436	49 (34–61)	1886	49 (35–61)
	N	%	N	%
**Men**	1086	44.6	842	44.6
**Women**	1350	55.4	1044	55.4
	2436	100	1886	100
**Place of residence**				
Town	2005	82.3	1886	100
Village	431	17.7	–	–
	2436	100	1886	100
**Household Asset Score**				
0–2	476	19.5	320	17.0
3	357	14.7	272	14.4
4	498	20.4	361	19.1
5	650	26.7	506	26.8
6	455	18.7	427	22.6
	2436	100	1886	100
**Highest attained education**^a^				
Primary school	1128	49.7	763	43.0
Upper secondary school	85	3.7	73	4.1
Short vocational (< 2 years)	741	32.6	642	36.2
Medium long (2–3 years)	290	12.8	270	15.2
Long, university(4+ years)	26	1.1	25	1.4
Missing	90	–	57	–
	2360	100	1830	100
**Job category**^b^				
Not working	344	18.0	239	16.2
Students	106	5.5	97	6.6
Unskilled labourers	692	36.2	497	33.7
Hunters/fishermen and families	113	5.9	51	3.5
Skilled workers	428	22.4	382	25.9
White collar employees	229	12.0	207	14.1
Missing	36	–	32	–
	1912	100	1505	100
**Food security**				
Secure	2197	90.3	1711	90.7
Insecure	235	9.7	175	9.3
Missing	4	–	–	–
	2436	100	1886	100

^a^ 18+ year old participants

^b^ 18–65 year old participants

The distribution of food insecurity in the population seen in Table 3. Men and young participants more often reported food insecurity and so did participants from households without children. There was a statistically significant indirect association between social position and food insecurity with 20% food insecure among the least wealthy and 0.4% among the wealthiest (*p* < 0.0001). Education and job category showed the same pattern with the highest prevalence of food insecurity among participants with only basic school education and among those who were not on the labour market. In Table 4, analyses were performed with adjustment for age and sex and with adjustment for age, sex and social position in univariate general linear models.

**Table 3:** Food insecurity by social and demographic variables. Greenland Population Health Survey 2018. *N* = 1886. Adjustment for age and sex in general linear models

	Food insecure	
	N	%
**Age group**		
15–24	242	19.3
25–34	226	13.2
35–59	899	9.1
60+	519	3.3
Total	1886	p < 0.0001
**Men**	842	10.9
**Women**	1044	7.9
Total	1886	*p* = 0.025
**Household**		
Households with children 0–17 year old	785	7.2
Households without children	1101	10.8
Total	1886	*p* = 0.018
**Household Asset Score**		
0–2	320	20.3
3	272	17.9
4	361	8.8
5	506	5.5
6	427	0.4
Total	1886	p < 0.0001
**Highest attained education**^a^		
Primary school	763	12.5
Upper secondary school	73	5.0
Short vocational (< 2 years)	642	7.0
Medium long (2–3 years)	270	3.6
Long, university(4+ years)	25	0.5
Total	1773	p < 0.0001
**Job category**^b^		
Not working	239	28.1
Students	97	12.9
Unskilled labourers	497	9.8
Hunters/fishermen and families	51	5.3
Skilled workers	382	5.5
White collar employees	207	3.4
Total	1473	p < 0.0001

^a^ 18+ year old participants

^b^ 18–65 year old participants

**Table 4:** Dietary patterns and cost of commodity basket by food security. Greenland Population Health Survey 2018. Population in towns. N = 1886. Adjusted for age, sex and social position in univariate general linear models and ratios between adjusted values for food insecure and food secure participants with 95% confidence intervals (CI)

	Adjusted for age and sex				Adjusted for age, sex and social position			
	Food secure	Food insecure			Food secure	Food insecure		
	%	%	Ratio food insecure: food secure	95% CI	%	%	Ratio food insecure: food secure	95% CI
**Dietary Pattern**								
Balanced diet	9.4	6.6	0.70	0.24–1.19	9.1	8.8	0.97	0.48–1.49
Kalaalimernit	5.4	3.7	0.69	0.07–1.35	5.4	3.8	0.70	0.09–1.37
Imported meat	5.4	3.0	0.56	−0.06-1.21	5.3	3.3	0.62	−0.01-1.30
Unhealthy	32.0	42.7	1.33	1.11–1.57	32.3	39.5	1.22	1.00–1.46
Standard diet	47.9	44.0	0.92	0.76–1.08	47.8	44.5	0.93	0.77–1.10
**Specific food groups**	g/day	g/day			g/day	g/day		
*Kalaalimernit* (marine mammals, birds)	72.9	57.3	0.79	0.64–0.93	72.7	59.9	0.82	0.68–0.97
*Kalaalimernit* (fish)	55.5	54.1	0.97	0–78-1.18	55.4	55.7	1.01	0.81–1.21
Fruit	171.3	89.1	0.52	0.35–0.70	168.8	114.2	0.68	0.50–0.86
Vegetables	122.1	82.5	0.68	0.54–0.82	120.7	96.1	0.80	0.65–0.94
**Weekly expenditure on commodity basket**^b^	DKK	DKK			DKK	DKK		
Nutritious food items	360	323	0.90	0.83–0.96	358	341	0.95	0.88–1.02
Non-nutritious items^a^	159	179	1.12	1.00–1.25	160	175	1.10	0.97–1.23
Food total	519	501	0.96	0.90–1.02	518	515	1.00	0.93–1.06
Alcoholic beverages	124	233	1.88	1.59–2.20	126	210	1.66	1.38–1.97
Tobacco	127	186	1.46	1.27–1.66	129	167	1.29	1.10–1.49
Total expenditure	770	920	1.19	1.12–1.27	773	893	1.15	1.08–1.23
**Energy in daily diet**	kJ	kJ			kJ	kJ		
Nutritious food items	5700	5778	1.01	0.96–1.07	5692	5859	1.03	0.97–1.09
Non-nutritious items^a^	2105	2541	1.21	1.09–1.33	2115	2443	1.16	1.04–1.28
Food total	7805	8319	1.07	1.01–1.12	7807	8302	1.06	1.01–1.12
Estimated energy requirement	10,434	9170	0.88	0.85–0.91	10,396	9542	0.92	0.89–0.94
Energy density (kJ/g)	5.3	5.9	1.11	1.07–1.15	5.3	5.8	1.08	1.04–1.12
**Cost per 1000 kJ (excl. Coffee and tea)**	DKK	DKK			DKK	DKK		
Nutritious food items	9.05	8.10	0.90	0.86–0.93	9.02	8.44	0.94	0.90–0.97
Non-nutritious items^a^	8.50	7.35	0.86	0.80–0.93	8.48	7.60	0.90	0.83–0.96
Food total	9.00	7.98	0.89	0.86–0.92	8.96	8.31	0.93	0.90–0.96
**Anthropometry**								
Body mass index (kg/m2)	27.5	24.1	0.88	0.84–0.91	27.4	24.9	0.91	0.88–0.94
Obesity (BMI 30+) (%)	29.6	13.1	0.44	0.22–0.67	29.1	18.2	0.63	0.39–0.87

^a^ Sweets, sugared beverages, fruit juice, cakes, sugar, convenience snacks, fast food, tea and coffee

^b^ 1 EUR = 7.45 DKK

The dietary pattern of participants who reported food insecurity differed significantly from that of other participants (Table 4). In particular, those who reported food insecurity were more often classified as eating an unhealthy diet. The food insecure participants consumed less *kalaalimernit* other than fish, and less fruit and vegetables whereas the consumption of local fish was not different between food secure and food insecure participants. The total expenditure on food was similar for food secure and food insecure participants, but those who reported food insecurity spent more money on non-nutritious food and less on nutritious food. This was, however, only statistically significant in the models adjusted for age and sex only. Furthermore, the expenditure on alcohol and tobacco of those who reported food insecurity was considerably higher than that of those who did not report food insecurity. The total reported energy consumption was higher for the food insecure participants than for other participants in particular due to a 16% higher energy consumption from non-nutritious food. The diet of the food insecure participants was more energy dense than that of other participants and their food was cheaper per kJ. Food secure participants had higher body mass index and were more often obese than food insecure participants.

We further compared the consumption of the individual 47 food groups between food insecure and food secure participants (not shown in the table). Adjusted for age and sex in univariate general linear models, participants who reported food insecurity consumed significantly more pork, white bread, pasta, fizzy drinks, junk food, ready-made food and sugar added to tea and coffee than other participants (*p* < 0.0001 for all food items). They consumed significantly less fruit and vegetables (p < 0.0001), whale meat (*p* = 0.02), caribou (*p* = 0.007), dried meat and fish (*p* = 0.009), mutton (*p* = 0.006) and dairy products (*p* = 0.029).

Table 5 shows that more than half of the participants who reported food insecurity were jobless (59%) and 75% had a high expenditure on alcohol and tobacco, defined as spending more than the median of DKK 210 per week. Participants aged 18–65 years (i.e. of working age) were divided into four categories according to employment status and expenditure on alcohol and tobacco. Among those who were both jobless and spent more than DKK 210 per week on alcohol and tobacco, 24% reported food insecurity compared with 3.1% among those who worked and spent less on alcohol and tobacco. High vs. low expenditure on alcohol and tobacco was associated with food insecurity irrespective of job status and job status was associated with food insecurity irrespective of expenditure on alcohol and tobacco. Being out of job and/or spending more than the population median on alcohol and tobacco accounted for 91% of the reported cases of food insecurity.

**Table 5:** Food insecurity according to job status and relative expense on alcohol and tobacco. Estimates adjusted for age and sex in univariate general linear models. Population health survey in Greenland 2018; participants aged 18–65 years. N = 1505

	Low expenditure on alcohol and tobacco	High expenditure on alcohol and tobacco	All		
	% food insecure			Ratio high/low expenditure	p
Working	3.1	10.8	6.8	3.48	p < 0.0001
Jobless	12.1	24.1	18.9	1.99	0.001
All	5.7	15.8		2.77	p < 0.0001
Ratio jobless/working	3.90	2.23	2.78		
p	p < 0.0001	p < 0.0001	p < 0.0001		
	N (%) food insecure				
Working	14 (9%)	52 (32%)	66 (41%)		
Jobless	27 (17%)	69 (43%)	96 (59%)		
Total	41 (25%)	121 (75%)			

## Discussion

The diet of participants who reported food insecurity was found to be less healthy than that of other participants. They consumed more energy and their food was more energy dense. The expenditure on food was similar in the two groups but the expenditure of the food insecure was significantly higher on non-nutritious food, alcohol and tobacco, their expenditure on nutritious food lower, and the cost of food per kJ lower.

The data was geographically representative of all of Greenland and the study base made up more than 6% of the adult Inuit population. Although the Inuit in Greenland is an indigenous people with a cultural past as hunters, today they mostly live a life as wage earners in modern towns by and large comparable to the life in Scandinavia and other Western countries. In our sample, less than 4% of participants were hunters/fishermen. It is therefore reasonable to assume that the sample is representative of indigenous and coastal populations in the affluent Western countries.

The key variables in the study will be discussed first, followed by a more general discussion of the findings. The definition of food and nutrition security of the Food and Agriculture Organization of the United Nations (FAO) emphasises availability, accessibility and utilisation of food [2]. Numerous methods have been used to measure food security [40], mostly targeting the access aspect. In the present study, the household hunger scale [37] was used, which has been specifically developed for cross-cultural use. The household asset score has certain advantages over other measures of social position, in particular that it is defined for all participants irrespective of age and is normally distributed. Education was skewed with almost half of the participants having no education beyond school and only 1.3% having a higher education. Job category excludes students and old-age pensioners and is only defined for the age group 25–64. Household asset score as an indicator of social position has been used in several studies from Greenland [27, 29, 41]. It differs from information about personal income in that it is a measure of the economic conditions of the entire household and that it is a measure of what the income is spent on by ways of durable goods [29]. Similar measures have been used elsewhere in the Arctic [42]. A population health survey from 2005 to 2010 showed a significant association between household asset score and income reported by Statistics Greenland based on information from the tax authorities: the disposable income was DKK 103,500 per person in the lowest household asset category compared with DKK 195,900 in the highest category (unpublished results).

Information on diet was obtained by face-to-face interviews, which are usually considered more reliable than self-administered questionnaires. Bias in reporting of diet in food frequency questionnaires may be introduced by the inability to recall and synthesise a general diet over a long period. The questions did not specify a short recent time period and although the interviewers were instructed to ask about the usual diet over the last 12 months, a certain seasonal bias in particular concerning *kalaalimernit* must be assumed. However, interviews were evenly spread over the year. The question of social desirability may also introduce bias, which may vary among social groups [43]. The definition of dietary pattern that was used in the present paper has proven its usefulness as independent variable in the analysis of diabetes [44] and food basket expenditure [24].

Energy intake was calculated from published values of macronutrients in the food. The accuracy of the estimate is dependent on the calculated food intake and the correspondence of actual food eaten with food items in the published tables. The food tables used were Greenlandic and Canadian values for *kalaalimernit* [31] and recently updated Danish values for imported food [32]. These values are believed to correspond well with actual food in Greenland. After exclusion of participants with unrealistically low or high reported energy intake, the reported average energy intake was 79% of the calculated energy expenditure. Underreporting of total energy intake estimated by food frequency questionnaires is common, ranging from 4.6 to 42% [45], so the 20% underreporting of the present study was not unduly high. It must be noted that relative to our previous article based on the same data [24], the reference values for energy and macronutrients as well as the method for estimation of criteria for exclusion have been updated with newly published information. The number of participants as well as energy and expenses are therefore not identical, although very similar.

It was confirmed that food insecurity is inversely associated with socioeconomic position measured by household asset score, education and job. The social indicators are closely associated [29] and it is uncertain whether one of the indicators is more germane to the study of food security than the others.

As hypothesised, the food insecure participants consumed less fruit and vegetables whereas the result for *kalaalimernit* was differed according to the specific food items. The consumption of the relatively inexpensive fish was the same in the two groups whereas food insecure participants consumed less of the more expensive marine mammals and birds. The dietary pattern of food insecure participants was more often classified as unhealthy, they consumed more non-nutritious food, and the cost per 1000 kJ of their food was lower than that of other participants, which all points to a diet of lower quality. Our hypothesis that the energy intake was lower among the food insecure was not supported. The total energy intake of the food insecure was actually higher than that of other participants due to a higher consumption of energy from non-nutritious food. Also, the energy density of their diet was higher. Contrary to our hypothesis, the weekly expenditure on food was similar among the two groups but our hypothesis that the food insecure spent more money on alcohol and tobacco than other survey participants was supported.

Food insecure persons in Greenland ascribed their unhealthy diet to the high cost of nutritious food [22]. In our study, the price per kJ was markedly lower for non-nutritious food (DKK 8.24) than for nutritious food (DKK 13.39) but the difference in price was outweighed by the purchase of relatively more non-nutritious food.

Whereas the calculated energy requirement was lower among the food insecure than among other participants, the reported energy intake of the former was higher. The calculated energy requirement among the food insecure participants was lower predominantly because they weighed less than other participants (62.3 vs. 74.2 kg adjusted for age and sex; *p* < 0.0001). Their body mass index was also lower and fewer were overweight or obese. This is at variance with most other studies where food insecurity is directly associated with overweight and obesity [46]. At present, we do not have an explanation for this. There could be several reasons why the reported energy intake was higher among the food insecure. First, their energy consumption might actually be higher than that of other participants. Second, the food insecure participants reported an energy intake equivalent to 92% of their calculated requirement whereas other participants reported only 77% (*p* < 0.0001; adjusted for age and sex). The food insecure may have a less varied diet, which may be easier to report. There were indeed significant differences in ratios between reported and calculated energy among dietary patterns. Thus, participants eating an unhealthy diet reported 85% of their calculated requirement and those eating a *kalaalimernit* diet 81% compared with 73–76% among participants with other dietary patterns (p < 0.0001).

The food secure and food insecure participants were able to spend equal amounts of money on food and the results thus suggest that there must be other explanations for food insecurity than poverty. Several studies agree that income is an important factor for food choice especially among persons living in low-income, food insecure households [10–14], but attitudes toward food prices also influence food purchase decisions [47] and the potential influence of cost may be overestimated [48]. Other motives are important and individual priorities in food choice motives play a role in producing social disparities in diet [49]. It is therefore probable that the differences in diet and expenses between food secure and food insecure participants are explained by other mechanisms than purchase power. Food security can be construed as a proxy for social position and the literature about social position and health behaviour is therefore pertinent for the discussion of food security and the unhealthy dietary habits of food insecure participants. The following explanations outlined by Pampel and coworkers [26] seem plausible in the Greenland context. First, disadvantaged social position and inequality has been shown to induce stress and reduce the capacity to cope. Inequality does exist in Greenland which has a Gini coefficient of 35 [50], notably higher than the EU average of 31 [51], and persons who reported food insecurity did perceive inequality and marginalization [22]. Second, education increases the efficacy, problem-solving skills, ability to process information and locus of control needed to govern daily life and overcome obstacles to good health [26]. Although participants with food insecurity cloaked their explanations for their situation in high prices and lack of money, there was a distinct undercurrent of reduced ability to plan ahead [22]. Third, less educated persons may have limited knowledge of the harm of unhealthy behaviour and therefore have less motivation to adopt healthy behaviours [26]. This may particularly be the case for dietary advice, which in the public debate is often perceived as being complicated, ever changing, and often conflicting.

Table 5 is a different approach to explaining the possible roles of poverty and competing expenses on alcohol and tobacco on food insecurity. In total, 91% of the cases of food insecurity could be explained by unemployment and/or a high expense on alcohol and tobacco. This is in line with a qualitative study from Greenland where the overall narrative was one of lack of money to buy food while several participants admitted that another reason for their food insecurity was that they prioritised alcohol and tobacco over food [22]. Studies from other parts of the world (USA, Nepal) showed similar associations of food insecurity with alcohol and tobacco [52, 53]

### Strengths and weaknesses

Strengths and weaknesses pertaining to individual variables have been discussed above. The study is part of a long series of population studies about health and social issues that date back to 1993 and thus build on a sizable amount of knowledge. It is a general strength of the study that it is countrywide and includes a large percentage of Greenlandic Inuit and that, geographically, the whole country is represented. The data on diet and cost are unique to this study. It is the first countrywide study of food security in Greenland and the first one to include expenditure on food.

It is a limitation that information on the income of participants was not available. It is thus not known how big a proportion of the participants’ budget was used on food and how much was available for other things. The lack of data on food prices in the villages where 14% of the Inuit population lives is a further weakness. The recruitment of participants was random but with a participation rate of 52% the possibility of recruitment bias is another weakness. The participation rate was lowest among young adults and among men. Few did not participate because of illness (2.8% of the sample). More did not participate because the interviewers were unable to contact them (12%), and most did not participate because they chose not to (31%) [23]. In general, persons without employment and other socially vulnerable persons, including those with a risky pattern of alcohol and drug use, are underrepresented in population health studies. This has been shown also to be the case for Greenland but only moderately so [54]. If the sample is socially skewed, we would expect the proportion of food insecure participants to be underestimated, the true population average of food expenses to be lower and the overall dietary pattern to be unhealthier. However, the differences between food secure and food insecure participants would not necessarily be biased.

An average price per food item was used to calculate the cost of food for all participants, but this reduces the interpersonal variation in actual expenses. Individual priorities in food choice play an important role [49]. Searching for bargain prices may be an economic necessity for some and, for a given food item, there may be cheap variants of low quality and more expensive variants that only the wealthy can afford. This is not reflected in our data and may result in bias when comparisons are made between food insecure participants and other participants. A lack of *kalaalimernit* may contribute to the perception of food insecurity as suggested in a previous study [22] but the survey questions did not cover this aspect, having a narrow economic definition of food security. However, food insecure participants did eat less meat of marine mammals than other participants but the same amount of fish.

It is a weakness of all cross-sectional studies that the direction of a statistically significant association cannot be determined. We presumed a high expenditure on alcohol and tobacco to be a contributing cause of food insecurity, but it is also possible than food insecurity, inequality and systematic marginalization all contribute towards a high consumption of alcohol and tobacco. This could then lead to more food insecurity and marginalization in a vicious circle. Likewise, our premise was that food insecurity affects dietary patterns, but the reverse could in principle also be the case.

## Conclusions

In this study of Greenlandic Inuit from 12 towns in Greenland, food insecure participants reported spending the same amount of money on food than other participants. They spent more money on non-nutritious food than other participants and spent more on alcohol and tobacco. The food insecure participants also reported consuming more energy than other participants and their food was more energy dense. These results suggest that it is not only unemployment and lack of money to buy nutritious food that creates food insecurity in Greenland. As far as spending can be conceived as a function of prioritisation, food insecure participants gave higher priority to buying non-nutritious food, alcohol and tobacco than did food secure participants, which might be explained by lack of resources for planning, prioritising and living one’s daily life, as well as limited knowledge about food. However, these mechanisms have not been studied in the present study. There seems to be at least two population subgroups in Greenland with poverty and substance use, respectively, as the immediate determinants for food insecurity. The results are important for the design of interventions against food insecurity and unhealthy dietary patterns. Our research results are continuously being discussed with the Greenland Government and an important approach has already been outlined in the strategy for an improved childhood by the Greenland Government, which with a focus on families with children plans to improve the diet of pre-school children, to increase awareness about diet and to reduce the consumption of alcohol and tobacco among parents [55]. Further studies of proximate sociodemographic determinants for food insecurity, such as household conditions, regional differences, education and subsistence hunting and fishing are needed.

## Supplementary Information

**Additional file 1.**

## Data Availability

The dataset supporting the conclusions of this article is in the process of being made available through the Danish National Archives (https://www.sa.dk/en/research-researchers-research-service-the-danish-national-archives/use-the-danish-national-archives-survey-data/). Due to the sensitive nature of the data restrictions will apply.

## Abbreviations

CI: Confidence interval
DKK: Danish kroner
E %: Energy percent
EUR: Euro
IQR: Interquartile range
kJ: Kilojoule
