# Supplementary material for: Association of food insecurity with dietary patterns and expenditure on food, alcohol and tobacco amongst indigenous Inuit in Greenland: results from a population health survey
Source: BMC Public Health. 2021 Jun 8;21:1094. doi: 10.1186/s12889-021-11123-x (PMC8186081; doi:10.1186/s12889-021-11123-x)
Supplement: Supplementary file 1 — Additional file 1. [file 12889_2021_11123_MOESM1_ESM.docx]

**Association of food insecurity with dietary patterns and expenditure on food, alcohol and tobacco amongst indigenous Inuit in Greenland: Results from a population health survey**

Peter Bjerregaard^a^

Ingelise Olesen^b^

Christina Viskum Lytken Larsen^a,b^

^a^ Centre for Public Health in Greenland, National Institute of Public Health, University of Southern Denmark, Studiestræde 6

1455 Copenhagen K, Denmark

^b^ Institute for Nursing and Health Research, University of Greenland, P.0. Box 1061, Manutooq 1, 3905 Nuussuaq, Greenland

Corresponding author: Peter Bjerregaard, National Institute of Public Health, University of Southern Denmark, Studiestræde 6, 1455 Copenhagen K, Denmark

Email: [pb@sdu.dk](mailto:pb@sdu.dk)

**Supplementary material**

Questionnaire translated from the original Greenlandic and Danish questionnaires into the English language. Numbers refer to the question number in the complete questionnaire.

| **13. Which school education do you have?** *(tick the highest level obtained)*  School attendant ⬜ 1  8^th^ grade or less ⬜ 2  9^th^-12^th^ grade ⬜ 3  Completed high school or similar ⬜ 4 |
| --- |

| **14. Have you completed further education?**  Yes ⬜ 1  No ⬜ 2  Write the name of the education or the diploma or certificate that you have obtained |
| --- |

| **23. How many people live in your house or your flat?**  Children < 5 yrs Children 5-17 yrs  Adults 18-59 yrs Adults 60 yrs or above |
| --- |

| **16. Which of the following best describes your present occupational status?**    Work for salary (full-time) ⬜ 1  Work for salary (regular part-time) ⬜ 2  Work for salary (occasionally) ⬜ 3    Self-employed (other than hunter/fisherman) ⬜ 4  Hunter/fisherman ⬜ 5  Housework ⬜ 6    Retired/pensioner ⬜ 7  Unemployed ⬜ 8  Social welfare ⬜ 9  Student ⬜ 10  Other (specify): ⬜ 10  **What is your position or your job?** *(Be specific: Assistant nurse at the hospital, not just ”work at the hospital”. Shop assistant at KNI, not just “work at the store”)* |
| --- |

| **25. Which of the following things do you have in your home?**  Yes No  **[H37A]** a. Video/DVD ⬜ 1 ⬜ 2  **[H37B]** b. Computer/laptop/iPad/tablet ⬜ 1 ⬜ 2  **[H37C]** c. Refrigerator ⬜ 1 ⬜ 2  **[H37E]** d. Deep freezer, chest freezer ⬜ 1 ⬜ 2  **[H37F]** e. Microwave oven ⬜ 1 ⬜ 2  **[H37G]** f. Washing machine ⬜ 1 ⬜ 2  **[H37H]** g. Dishwashing machine ⬜ 1 ⬜ 2  **[H37M]** h. Internet/mobile internet ⬜ 1 ⬜ 2  **[H37I]** i. Dinghy or boat ⬜ 1 ⬜ 2  **[H37K]** j. Car, snow scooter or cross-country scooter ⬜ 1 ⬜ 2 |
| --- |

| **The next questions are about your diet** |
| --- |

| * d=number of times per day; u=number of times per week; m=number of times per month; å=number of times per year; 0=Not at all. |
| --- |

| **How often do you eat the following?** |  |  |
| --- | --- | --- |
| ***Marine mammals*** | *How often?* | *Portion size* |
| Seal meat |  |  |
| Whale meat |  |  |
| Mattak |  |  |
| **When you eat seal, whale or walrus, how much is your serving size?** |  |  |

| **How often do you eat the following?** |  |  | |
| --- | --- | --- | --- |
| ***Fish and land mammals*** | *How often?* | | *Portion size* |
| Cod |  | |  |
| Greenland halibut |  | |  |
| Capelin |  | |  |
| Trout, salmon |  | |  |
| Other fish |  | |  |
| **When you eat fish, how much is your serving size?** |  | |  |
| Caribou meat, muskox |  | |  |

| **How often do you eat the following?** |  |  |
| --- | --- | --- |
| ***Other*** | *How often?* | *Portion size* |
| Game birds |  |  |
| Berries |  |  |
| Dried fish or meat |  |  |
| Blubber (frozen, salted) |  |  |

| **How often do you eat the following?** |  |  |
| --- | --- | --- |
| ***Imported food products*** | *How often?* | *Portion size* |
| Beef |  |  |
| Pork, e.g. chops, roast pork, meat balls, sausages |  |  |
| Lamb (Greenlandic or imported) |  |  |
| Poultry (chicken, turkey, duck) |  |  |
| **When you eat these types of meats, how much is your serving size?** |  |  |
| Readymade dishes (canned, frozen) |  |  |
| Cold cuts, liver paste |  |  |
| Pickled herring, canned fish |  |  |

| **How often do you eat the following?** |  |  |
| --- | --- | --- |
| ***Fruit and vegetables*** | *How often?* | *Portion size* |
| Apples, pears, bananas |  |  |
| Oranges, grapefruit |  |  |
| Other fresh fruit |  |  |
| **When you eat fresh fruit, how much is your serving size?** |  |  |
| Fruit juice |  | *glasses* |
| Potatoes |  |  |
| Mixed vegetables/frozen vegetables |  |  |
| Carrots |  |  |
| Cabbage (e.g. white cabbage, red cabbage, cauliflower, broccoli) |  |  |
| **When you eat vegetables, how much is your serving size?** |  |  |
| Tomatoes |  |  |

| **How often do you eat the following?** |  |  |
| --- | --- | --- |
| ***Dairy products and bread*** | *How often?* | *Portion size* |
| Milk and milk products |  |  |
| Cheese |  |  |
| White bread |  | *Slices* |
| Ryebread |  | *Slices* |
| Cornflakes, Guldkorn, or other breakfast cereals |  |  |
| Oatmeal, hot or cold |  |  |
| Spaghetti, pasta |  |  |
| Rice |  |  |

| **How often do you eat the following?** |  |  |
| --- | --- | --- |
| ***Other*** | *How often?* | *Portion size* |
| Cakes, Danish pastries, biscuits |  |  |
| Sweets (chocolate bars, wine gums, liquorice, hard candies) |  |  |
| Fizzy drinks, coke |  | *bottles á ml* |
| Fruit syrup with water |  | *glasses* |
| Pizza, burgers |  |  |
| French fries |  |  |
| Crisps, chips |  |  |

| * d=number of times per day; u=number of times per week; m=number of times per month; å=number of times per year; 0=Not at all. |
| --- |

| **73. How many cups of coffee or tea do you drink during the day?**  **Coffee (total cups)**  How many sugar cubes or how many spoons of sugar do  you use per cup?  **Tea (total cups)**  How many sugar cubes or how many spoons of sugar do  you use per cup? |
| --- |

| **77. In the past 12 months, have there been times where there was no food in the house, nor money to buy food?**  Yes ⬜ 1  No ⬜ 2 |
| --- |

| **78. In the past 12 months, have there been times where you’ve gone to bed hungry because there was not enough food?**  Yes ⬜ 1  No ⬜ 2 |
| --- |

| **79. In the past 12 months, have you gone a whole day and night without eating anything because there was not enough food?**  Yes ⬜ 1  No ⬜ 2 |
| --- |

| **93.** **Do you smoke?**  Yes, daily ⬜ 1 → go to ques. 95  Yes, but some days I don’t smoke ⬜ 2 → go to ques. 95  No ⬜ 3 |
| --- |

| **94. Have you been smoking earlier?**  Yes ⬜ 1  No ⬜ 2 → go to ques. 99    ***If yes:***  **When did you quit smoking?** _____________year |
| --- |

| **95. How much do you smoke a day on average?**  *Previous smokers:*  **How much did you smoke a day on average?**   1. number of cigarettes a day |
| --- |

| **S28. How often do you consume something that contains alcohol?**  Never ⬜ 1 →go to quest. 40  No more than once a month ⬜ 2  2-4 times a month ⬜ 3  2-3 times a week ⬜ 4  4 times a week or more ⬜ 5 |
| --- |

| **S29. How many drinks do you usually drink when you do drink?**  1-2 drinks ⬜ 1  3-4 drinks ⬜ 2  5-6 drinks ⬜ 3  7-9 drinks ⬜ 4  10 or more drinks ⬜ 5 |
| --- |
